# A Systematic Review of the Literature on Parenting of Young Children with Visual Impairments and the Adaptions for Video-Feedback Intervention to Promote Positive Parenting (VIPP)

**DOI:** 10.1007/s10882-016-9529-6

**Published:** 2016-12-07

**Authors:** Ellen G. C. van den Broek, Ans J. P. M. van Eijden, Mathilde M. Overbeek, Sabina Kef, Paula S. Sterkenburg, Carlo Schuengel

**Affiliations:** 1 Royal Dutch Visio, Amersfoortsestraatweg 180, 1272 RR Huizen, The Netherlands; 2 Faculty of Behavioural and Movement Sciences, Clinical Child and Family Studies and the EMGO+ Institute for Health and Care Research, Vrije Universiteit Amsterdam, Van der Boechorststraat 1, 1081 BT Amsterdam, The Netherlands; 3 Bartiméus, P.O. Box 87, 3940 AB Doorn, The Netherlands

**Keywords:** Visual impairment, Parent-child relationship, Development, Visual-and-intellectual disability, Intervention

## Abstract

Secure parent-child attachment may help children to overcome the challenges of growing up with a visual or visual-and-intellectual impairment. A large literature exists that provides a blueprint for interventions that promote parental sensitivity and secure attachment. The Video-feedback Intervention to promote Positive Parenting (VIPP) is based on that blueprint. While it has been adapted to several specific at risk populations, children with visual impairment may require additional adjustments. This study aimed to identify the themes that should be addressed in adapting VIPP and similar interventions. A Delphi-consultation was conducted with 13 professionals in the field of visual impairment to select the themes for relationship-focused intervention. These themes informed a systematic literature search. Interaction, intersubjectivity, joint attention, exploration, play and specific behavior were the themes mentioned in the Delphi-group. Paired with visual impairment or vision disorders, infants or young children (and their parents) the search yielded 74 articles, making the six themes for intervention adaptation more specific and concrete. The rich literature on six visual impairment specific themes was dominated by the themes interaction, intersubjectivity, and joint attention. These themes need to be addressed in adapting intervention programs developed for other populations, such as VIPP which currently focuses on higher order constructs of sensitivity and attachment.

Children with visual impairments face multiple problems in the development of adaptive functioning. Children need to learn how to understand the physical world in order to acquire cognitive skills, and to perceive and understand social relationships (Warren 1994). These developmental issues are more complicated when vision is lacking or impaired, given the heightened stress for children as well as for primary caregivers. For many parents, having a child with a disability already requires dealing with guilt, blame, or reduced self-esteem as well as disappointment, sadness, depression (Neely-Barnes and Dia 2008; Reichman et al. 2008). Furthermore, many children with visual impairments have additional impairments depending on the underlying cause of their disability. For example, extreme prematurity or a syndrome can lead to intellectual and functional disabilities (Volpe 2009). A study in Nordic countries indicated that almost two thirds of children with a severe visual impairment had additional impairments (Rosenberg et al. 1996). The strain on the parent-child relationship (Howe 2006) may manifest itself in lowered parental sensitive responsiveness and insecure attachment. Sensitivity and attachment are highly relevant targets for preventive intervention because children growing up in secure attachment relationships have a better quality of life and sensitive parenting and secure attachment may increase the family’s resilience against the challenges associated with impairment as well as facilitate caregiving and education (Guralnick in press).

Sensitive responsiveness is an important concept in attachment theory (Bowlby 1969; Ainsworth 1973). Children are born with the tendency to seek contact and proximity to familiar caregivers, especially when danger is perceived. The nature of the response of the caregivers therefore carries an important affective meaning, explaining why caregiving characterized by responsiveness and sensitivity to children’s signals and needs is associated with patterns of child attachment behavior characterized by open communication and effective use of the caregiver as a source of comfort. These children learn to use their caregiver as a secure base from which to explore the world and as a safe haven to return to in times of distress. For parents of a child with a visual or visual-and-intellectual disability interacting in a sensitive and appropriate way may be more difficult as the child may seek proximity through different behavior than a sighted child would (Fraiberg 1975). Other studies however indicate that the attachment relationships of parents and children with visual impairments do not differ significantly from those of sighted children (Gerra 1993; Friedman 1988).

Rearing a child with a visual impairment can be stressful for parents (Tröster 2001). Early intervention with a focus on parent child interaction can help to relieve this strain. A secure attachment relationship between parents and children may diminish the negative consequences of a visual impairment for the development of the child. Since 1980 parents have been able to consult early intervention centers that serve the needs of parents and their children with visual impairments (Van Dijk 2002). Intervention to promote a secure attachment relationship, to support the understanding of specific aspects of the child’s behavior and to strengthen sensitive responses of parents, may benefit the development of children with disabilities (Schuengel et al. 2013). A meta-analysis on interventions that focus on parental sensitivity and quality of parent-child attachment relationships indicated that short-term interventions with a focus on sensitive parenting are more effective than long-term (more than 16 sessions) interventions with a broad focus (Bakermans-Kranenburg et al. 2003). Video-feedback Intervention to promote Positive Parenting (VIPP) (Juffer et al. 2008a) is an intervention based on precisely this blueprint, involving 8 home visits using video to improve quality of attachment by focusing on sensitive parenting. The focus is on 1) exploration versus attachment behavior, showing the difference between contact seeking behavior and play behavior, 2) ‘speaking for the child’; promoting accurate perception of the signals of the child, 3) ‘sensitive chain’ explaining the relevance of adequate responding to the signals and 4) sharing emotions (Juffer et al. 2008b). Parents are supported to closely follow their child; to notice the signals of their child, interpret them and respond to them in a sensitive way. The VIPP program has since then been adapted for several populations, including parents of children with challenging behavior (VIPP-SD; Van Zeijl et al. 2006, Juffer et al. 2016), parents of children with autism (VIPP-AUTI; Poslawski et al. 2014) and parents with a learning disability (VIPP- LD; Hodes et al. 2014) and tested in randomized controlled trials. The current review is a step towards developing adaptations to meet the needs of parents of children with visual or visual-and-intellectual impairments. However we believe that the findings are also relevant for adapting other effective intervention programs to improve parental sensitivity and quality of attachment.

In this study the two research questions were: 1) What themes need to be changed to adjust the VIPP to VIPP-V (Video-feedback Intervention to promote Positive Parenting for parents of children with visual or visual-and-intellectual impairments) and to adjust interventions for children with visual or visual-and-intellectual impairments; 2) What are the broader implications for practice concerning the stimulation of the development of a child with a visual or visual-and-intellectual impairment and the improvement of parent-child attachment?

## Method

A Delphi-group consisted of professionals representing all early intervention departments of Royal Dutch Visio and Bartiméus in The Netherlands. Clinical child psychologists and early interventionists in the field of family support for children with visual impairments (*N* = 13) participated. First, the most commonly used literature (e.g. Dik 2005; Fraiberg 1977; Gringhuis 1996; Warren 1994) and videos with interaction between parent and a child with a visual or visual-and-intellectual disability were discussed using the question: ‘How would we talk to the parent of a child with a visual impairment using the VIPP approach?’ In a second round, themes were identified which are important in supporting parents of a child with a visual impairment. During the third round the group was divided into three sub-groups. Each sub-group addressed the central question ‘Which are the most important themes for VIPP-V?’. After having identified the most important themes in the three sub-groups, the participants reached plenary consensus on the themes. Finally, the themes were discussed with the VIPP-developers at Leiden University. The themes were then the keywords in the literature search. The purpose of the literature review was to substantiate the themes reported in the Delphi search, to find more detailed subthemes and to advise on the content of the definitive manual of the VIPP-V.

A systematic literature search was performed between February 2014 and August 2014, using PsychInfo, Wiley online library, the index of the Journal of Visual Impairment and Blindness, the EBSCOhost databases and Pubmed. Also, hand selected searches were done up to August 2014 by examining the reference sections of the articles found in the automatic search process, the review studies and chapters in books on the topic of development, behavior and parent-child interaction in children with visual impairments. Key words used in the search were: visual impairment or vision disorders, infants or young children (and their parents) paired with the themes advised by the Delphi-group: interaction/communication, inter-subjectivity, joint attention, exploration, play behavior, and specific behaviors. The inclusion criteria were: a) studies focusing on children with visual impairments or children with visual impairments in combination with intellectual and/or multiple disabilities; b) studies based on empirical studies on children aged zero up to six years or if the children under six years were clearly defined as a separate age group; c) no limits to size of sample; d) articles published in a peer reviewed journal; e) articles published between 1968 (the year in which Fraiberg described the development of 10 infants with blindness) and February 2014; f) articles in all languages were included. The exclusion criteria were: a) books; b) dissertations; c) review articles without additional insights; d) articles on visual impairment of the parent instead of the child; e) articles on assessment of visual impairment and effectiveness of treatment of the impairment; f) articles on teacher-child interactions and peer interactions instead of parent-child interactions; g) articles on visual functioning in children with specific visual impairments/syndromes.

The Delphi-consultation identified six themes that were deemed important for the adjustment of VIPP to VIPP-V. These themes were: Interaction/communication, Intersubjectivity, Joint attention, Exploration, Play and Specific behaviors. These themes were then defined as follows:

- Interaction/communication: This concept refers to reciprocity and sharing of interests and emotions by non-verbal and verbal behaviors. There is mutual influencing: the signals are sent, interpreted and responded to by child and parent. Thus, learning to communicate is learning to make ones intentions known and learning how to recognize the intentions of others (Bruner 1984). For the theme interaction/communication articles were included which described the interaction between both partners; the repertoire of signals used, and the interpretation of and response to these signals.
- Intersubjectivity: In the early interaction between infant and caregiver, intersubjectivity is a core process (Loots et al. 2003). Intersubjectivity is described as the reciprocal exchange or ‘dialogue’ between infant and caregiver. Rudimentary forms of intersubjectivity exist in this dyadic interaction immediately after birth. During the first weeks, expressions and reactions of infant and parent become more and more attuned; infant and parent imitate one another’s behavior and share emotional experiences. Trevarthen (1979) describes both neonatal imitation and mutual regulated communication from two weeks of age. Infants can recognize contingency in reactions of their parents which makes protoconversations, subtle and reciprocal coordinated vocal and behavioral dialogues, possible. Articles were included for intersubjectivity that described dyadic early interaction and communication before speech.
- Joint attention: This concept refers to the intersubjective sharing of two persons that focus their attention on the same object or activity and that are both aware of their mutual interest (Tomasello 1995). Joint attention is a triadic interaction (subject, subject, object); in pointing and following a finger or gaze one not only relates to the object itself, but also to the other person’s feelings and interests for this object. Joint attention arises out of developments in infants’ social interactions on the one hand and their interactions with objects on the other hand (Bigelow 2003) and in sighted children emerges towards the end of the first year (Tomasello 1995). Lack of vision might hinder the development of a Shared Attention Mechanism (SAM) although SAM can use information from any modality (also touch, hearing) to determine another’s focus of attention (Baron-Cohen 1994). Articles on joint attention were included if the focus in the article was on triadic interaction: subject, subject, object.
- Exploration: Exploration is the tendency to examine and investigate a novel environment and overlaps with curiosity. Exploration is dependent on sensory and neuro-muscular resources and enables children to discover the possibilities in their surroundings (Gibson and Schumuckler 1989; Gibson 1979). Orienting, reaching, and grasping are embedded in the search for information, and are enabled by a state of awareness about the self and its environment (Smitsman and Schellingerhout 2000). Articles focusing on exploration were included if they described the quantity and quality of exploration and locomotion.
- Play: Play refers to a range of voluntary, intrinsically motivated activities associated with pleasure and enjoyment which are initiated and controlled by the child. The behavior is purely self-contained and serves no other purpose than joy (Hellendoorn and Berckelaer-Onnes van 1991; Hellendoorn et al. 1994). Play is not just a pleasurable activity; it stems from children’s eagerness to learn about the world and wanting to be part of it (Moleman et al. 2011). Play evolves from the stage of manipulating objects to relational, functional and symbolic play (Hellendoorn 1989; McCune-Nicolich 1980). Articles were included under the theme ‘play’ if the study described the quantity or quality of play or the development of play.
- Specific behaviors: Stereotyped movements and behaviors e.g. body rocking, head shaking, eye poking and hand flapping often occur in children with visual impairments and are sometimes called ‘blindisms’ (Fazzi et al. 1999). Stereotyped behaviors are defined as any repetitive or stereotyped movement, which is not directed towards the attainment of any observable obvious goal (Eichel 1978). Studies on quality or quantity of specific movements and behaviors often seen in children with visual impairments were included.

**Fig. 1:**
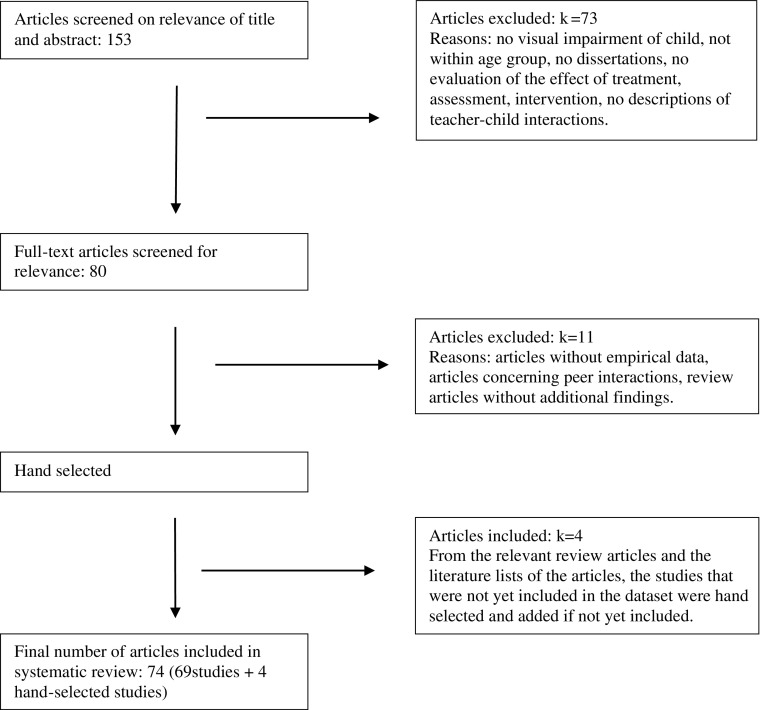
Diagram of the process of selection of literature for the systematic review

The selection process for the literature review was as follows (see Fig. 1): First, titles and abstracts identified by the electronic search (*k* = 153) were matched with the inclusion criteria. When the abstracts did not provide sufficient information regarding the criteria, full text evaluation was carried out. Second, of the remaining abstracts full text copies (*k* = 80) of the articles were examined and 11 articles were excluded (*k* = 69). Finally, five hand-selected articles were added (*k* = 74). Although articles in all languages were included, the number of non-English articles was very small as the search terms were in English.

All included studies (*k* = 74) were reviewed by the first two authors separately and coded on the six themes mentioned in the Delphi-search (Table 1). Most articles could be coded under more than one theme. Inter-rater agreement was sufficient to good with Kappa’s ranging from .60 to .89. The inter-rater reliability for the articles on Exploration was .36, therefore the first two authors both read all selected articles on Exploration again and reviewed coding until consensus was reached.

**Table 1:** Summary of articles indicating sample, design, instruments, and (sub)themes (*k* = 74)

Authors, year of publication and origin	Sample size sample age	Impairment/no impairment	Design	Instruments	(Sub)themes
Als et al. (1980) USA	*N* = 1 Age: at start 10 d. - 15 ½ m.	Blind*, no additional impairments**	Longitudinal descriptive design	Brazelthon behavior assessment Video observation of face to face interactions and object play	Interaction^3^ Intersubjectivity^1,2,3^ Joint attention^1^ Exploration^1^ Specific behavior^3,4^
Andersen et al. (1984) USA	Bl: *N* = 4 Si: *N* = 2 Age: 0.9 - 3.4 y.	Blind, sighted	Longitudinal descriptive design	Video, audio analysis on the acquisition process of language	Interaction^3^ Joint attention^2^ Exploration^3^ Play behavior^2,3^
Baird et al. (1997) USA	VI: *N* = 7 Age: 4–22 m. Si: *N* = 60 Age: 2½ -7 m.	Visually impaired, additional impairments	Comparative descriptive design	Video analysis mother child interaction. Mothers were asked to identify meaningful behaviors	Interaction^1,3^ Intersubjectivity^3^
Behl et al. (1996) USA	VI: *N* = 24 (dyads) Bl: *N* = 7 (dyads) Si: *N* = Gr I 31(dyads) *N* = Gr II 31(dyads) Age: 15–61 m.	Visually impaired, blind, sighted with mild additional impairments	Comparative observational design VI group and two comparison groups of sighted children with mild delay. One com group for each of the two rating systems.	Video analysis mother child dyad: free play Two standardized rating systems: parental behavior rating scale and parent/caregiver involvement scale.	Interaction^2^
Bigelow (1992) Canada	*N* = 3 Age at start: 13, 17, 32 m. Age at end: 26, 42, 45 m.	Blind, no additional impairments	Longitudinal observational design	Once a month observation at home, parents interview about new motor behavior: correlation between locomotion and object search.	Exploration^1,2^
Bigelow (2003) Canada	*N* = 2 Age 1: 13 - 23 m. Age 2: 21 - 30 m.	Blind, no additional impairments	Longitudinal observational design	Monthly video-analysis. Behavior relevant to the emergence of joint attention.	Intersubjectivity^2^ Joint attention^1^ Exploration^1,2^ Play behavior^2^ Specific behavior^2,4^
Brambring and Tröster (1992) Germany	*N* = 52 Two age groups *N* = 24: 7–24 m. *N* = 28: 25–56 m.	Blind, no additional impairments (premature)	Comparative descriptive design between two points in time.	Bielefelder questionnaire stereotyped behavior, two points in time: interval average 15,7 m (range 11-20 m.) Sample divided in two age groups.	Specific behavior^1,2,4^
Brambring (2005) Germany	*N* = 1 Age: 36 m.	Blind, no additional impairments	Observational single case study	Video analysis testing sequence perceptual perspective taking.	Interaction^3^ Joint attention^1^
Brambring and Asbrock (2010) Germany	Bl: *N* = 45 Age: 4–10 y. (*N* = 14 4–5 y.) Si: *N* = 37 Age: 3–6 y.	Blind, no additional impairments	Comparative design	First order false belief tasks Tom tasks based on tactile and auditory experience.	Interaction ^3^ Joint attention^1^
Campbell (2003) Australia	Bl: *N* = 4 Si: *N* = 4 Age: 18 m.	Blind, no additional impairments (2 premature), sighted	Observational comparative descriptive design.	Video analysis of play sessions mother-child Child Data language exchange system: mother’s and child’s utterances and actions: frequency and length.	Interaction^2^ Joint attention^3^ Specific behavior^4^
Campbell (2007) Australia	*N* = 2 (dyads) Age: 19 m.	Blind, no additional impairments	Observational descriptive design of two case studies	Quality of interaction in play context, using the Emotional Availability Scale (EAS).	Interaction^3^
Celeste (2006) USA	*N* = 1 Age: 4.6 y.	Visually impaired, no additional impairments	Observational single case study	Play behavior and social interactions. Developmental and adaptive assessments BDI, Oregon, VABS, structured play assessment, peer related social behavior, POS, ISBS sociometric ratings, interviews	Exploration^1^ Play behavior^3^
Conti-Ramsden and Perez-Pereira (1999) Spain	*N* = 1 Bl (dyad) *N* = 1 VI (dyad) *N* = 1 Si (dyad) Age: 22 m.- 23-25 m.	Blind (premature), visually impaired, sighted	Comparative design	3 monthly video analysis of conversational interactions of mother child dyads: Verbal and nonverbal events. CHAT and CHILDES	Interaction^1,2^ Joint attention^3^
Dale and Sonksen (2002) UK	*N* = 69 Age Time 1: 10–16 m. Age Time 2: 27–54 m.	Visually Impaired, blind (PVI no form vision), no additional impairments	Comparative study at time 1 and time 2	Visual assessment and developmental assessment	Interaction^1^ Joint attention^1^
De Campos et al. (2012) Brazil	Review *N* = 18 studies Age: 4–24 m.	Children at risk	Review	Role of developmental risk factors on development of exploratory actions.	Exploration^2^
Demingeon-Pessonneaux et al. (2007) France	*N* = 9 (dyads: mother- child, stranger- child) Age: Si: *N* = 3 5–8 m. VI: *N* = 3 (1 Bl) 12–41 m. VI+ add imp: *N* = 3 22–62 m.	Sighted, visually impaired (1blind), visually impaired with additional impairments	Quantitative computer program registries interaction verbal, non-verbal	Quantitative computer analysis of interactions and communication Dynamic lnterCoder System	Interaction^3^
Dote-Kwan and Hughes (1994) USA	*N* = 18 (dyads) Age: 20–36 m.	Blind, no additional impairments (7 premature)	Descriptive correlational design	Relation HOME factors and Reynell-Zinkin scale and Maxfield Buchholz scale ,relation SES and Reynell-Zinkin scale and Maxfield Buchholz scale	Interaction^2^
Dote-Kwan (1995) USA	*N* = 18 (dyads) Age: 20–36 m.	Blind, no additional impairments, (7 premature)	Observational correlational design	Impact of mothers- child interaction on the development of child. M-C scale, Reynell Zinkin scale, Maxfield Buchholz scale	Interaction^2^
Dote-Kwan et al. (1997) USA	*N* = 15 (dyads) Age (Time 1: 20–36 m.) Age (Time 2: 32-48 m.)	Blind and visually impaired, no additional impairments	Descriptive correlational design	Mothers behavior, early home environment and development of child. HOME, M-C interaction rating scale	Interaction^2^
Farrenkopf and Davidson 1992 Canada	Bl: *N* = 21 Age: 3–8 y. (*N* = 7: 3–4 y.) Si: *N* = 60 ( 30 blind folded, 30 sighted) Age 3–8 y.	Blind, no additional impairments, sighted, with and without blindfold	Comparative study design	Comparing perspective taking abilities in blind, blind folded and sighted child Perspective taking test.	Interaction^3^ Joint attention^1^
Fazzi et al. (1999) Italia	Bl no add imp *N* = 10 Bl add imp *N* = 16 Age: 4–60 m.	Blind without additional impairments, Blind with additional impairments	Descriptive design	Video analysis and questionnaire	Interaction^1^ Specific behavior^2,3,4^
Ferguson and Buultjens (1995) UK	*N* = 16 Age: 1.4–6.2 y.	Blind, no additional impairments (5 premature)	Two years longitudinal exploratory study, descriptive design	Monthly video analysis of play, correlation between play categories and age and scores on Reynell Zinkin scales.	Interaction^1^ Exploration^1,2,3^ Play behavior^1,2^ Specific behavior^3,4^
Fraiberg (1968) USA	*N* = 8 Age: 3 d. to 6 m. (at start) 18 m.– 6 y.	Blind, no additional impairments	Longitudinal observational design	Biweekly video analysis of mother child interaction, development child Developmental tests (Cattell, VABS, Maxfield Buchholz scale)	Interaction^1,3^ Intersubjectivity^1,2,3^ Exploration^1,2,3^ Specific behavior^2,3^
Fraiberg and Adelson (1973) USA	*N* = 4 Age: 9 m.- 6 y. (1 subject detailed)	Blind, no additional impairments (3 premature)	Longitudinal descriptive study.	Biweekly narrative description and video analysis of mother child interactions	Interaction^1^ Play behavior^1,2^
Fraiberg (1975) USA	*N* = 10 Age: at start 1–11 m. - 24 m.	Blind, no additional impairments (3 premature)	Longitudinal observational design	Bimonthly video analysis, describing characteristics of attachment behavior	Interaction^1^ Intersubjectivity^1,2^ Exploration^1,2^
Freeman et al. (1989) Canada	Bl: *N* = 92 Si: *N* = 92 Age: < 2–19 y. (*N* = 11 < 4y.)	Blind without and blind with additional impairments, sighted	Longitudinal quantitative descriptive prevalence study 1973 and follow up in 1987 (*N* = 82)	Parents questionnaires, semi structured interviews, school questionnaires, psychological pediatric, neurological assessment	Specific behavior^1^
Gense and Gense (1994) USA		Blind with and without autistic features	Review	Comparing stereotypic behavior in children with autism and children with VI	Specific behavior^1^
Gerhardt (1982) USA	*N* = 3 detailed longitudinal info on 1 child at Age: 14, 16, 18 m.	Blind, no additional impairments	Descriptive design, longitudinal data (*N* = 1)	Video analysis of play sessions with 8 different set of tasks Focus: development of object play and classificatory skills.	Exploration^1,2^ Play behavior^3^
Hobson et al. (1999) UK	Bl: *N* = 9 Si: *N* = 9 age: 5–9 y.	Blind with ASD, sighted with ASD	Quantitative design	Comparison on rating scales, (CARS, BCDP,) Interview parents	Interaction^1^ Joint attention^1^ Exploration^3^ Play behavior^3^
Hughes et al. (1999) USA	VI: *N* = 9 (dyads) Bl: *N* = 8 (dyads) Age: 20–36 m.	Visually impaired, blind no additional impairments (6 premature)	Observational correlational design:	Amount, quality and appropriateness of maternal behaviors rated in play sessions compared with language development of children with VI	Interaction^2^
Jan et al. (1983) Canada	congenital Bl: *N* = 512 acquired Bl: *N* = 107 Age: 0–18 y.	Blind	Retrospective descriptive design	Comparing visual state, age, IQ and stereotypic behavior	Specific behavior^1,2^
Kekelis and Andersen (1984) USA	Bl: *N* = 2 VI: *N* = 2 Si: *N* = 2 Age: 1–3 y.	Blind, visually impaired, no additional impairments, sighted	Comparative descriptive design	Video audio analysis and detailed transcriptions of parent child interaction.	Interaction^2^ Joint attention^3^ Specific behavior^3^
Kekelis and Prinz (1996) USA	Bl: *N* = 2 Si: *N* = 2 Age: 27–36 m.	Blind, no additional impairments. sighted	Comparative descriptive design	Monthly video analysis of mother child play sessions	Interaction^1,2^ Joint attention^2,3^ Play behavior^3^
Kreutz and Bosa (2009) Brazil	Age: preschool	Visually impaired and blind	Review	Review of early parent child interaction. Review of efficacy of early intervention.	Interaction^3^
Landau (1991) USA	Four experiments Experiment 1: Bl: *N* = 3 Si: *N* = 16 Experiment 2–4: Bl: *N* = 1 Age: 18–36 m.	Blind (1 premature), sighted	Experimental design	Haptic exploration observation and spatial representation tasks	Exploration^1^
Lappin and Kretschmer (2005) USA	*N* = 1 Age: 11 m.	Visually impaired (premature)	Observational and descriptive intervention design	Mother child interaction before and after a protocolled infant massage intervention.	Intersubjectivity^2^
Lewis et al. (2000) UK	Bl: *N* = 12 VI: *N* = 6 Age: 21–86 m.	Blind, visually impaired, (4 with criteria for ASD)	Observational descriptive design	Structured play tests and development assessments (TOPP, SPT, CARS, Reynell Zinkin scale).	Interaction^1^ Exploration^3^ Play behavior^3^
Loots et al. (2003) Belgium		Visually impaired	Review	Intersubjective developmental theory used to integrate the seemingly incoherent research findings on interactions	Intersubjectivity^1^ Joint attention^1^
Mallineni et al. (2006) India	Bl: *N* = 3 VI: *N* = 9 Age: 2–8 y.	Blind, visually impaired ( 10 additional impairments)	Observational descriptive design	Video analysis of nonverbal behavior	Interaction^3^
McConachie and Moore (1994) USA	Bl: *N* = 9 VI: *N* = 9 Age: 10–20 m.	Blind, visually impaired (2 additional impairments)	Descriptive comparative design	6–8 m. interval assessments (Reynell Zinkin scale) and diary of emerging words, language milestones and content.	Interaction^1^ Exploration^2,3^
de Medeiros and Salomão (2012) Brazil	*N* = 3 (dyads) Age: 6–20 m.	Blind, (premature)	Observational descriptive design	Bimonthly video analysis of mother child interaction in play situation Descriptions of interactive episodes	Interaction^3^ Intersubjectivity^3^ Joint attention^3^
Moore and McConachie (1994) USA	Bl: *N* = 8 VI: *N* = 8 Age: 15–25 m.	Bind, visually impaired, no additional impairments	Observational comparative descriptive design	Video analysis of parent child interaction in play situation.	Interaction^2^ Joint attention^3^
Olson (1983) USA	Bl: *N* = 15 Si: *N* = 15 Age: 2.1–6.3 y.	Blind, no additional impairments, sighted	Observational descriptive comparative design	Video analysis of exploratory behavior comparing blind child and sighted child approaches to two types of toys.	Exploration^3^
Ophir-Cohen et al. (2005) Israel	*N* = 74 Age: 6–59 m.	Visually impaired	Descriptive correlational design	Emotional, behavioral deficit defined by clinical assessment of emotional and behavioral deficits, developmental assessments and mothers education	Interaction^1,3^ Exploration^2^
Parr et al. (2010) UK	*N* = 83 Age:10 m. – 6.10 y.	Blind, visually impaired	Retrospective descriptive design	Vision assessment, development assessment, clinical judgment of social communication and repetitive restricted behavior, ASS and child with ONH and SOD	Interaction^1,3^ Specific behavior^1^
Parsons (1986a) UK		Blind, visually impaired	Review	Case studies and empirical research of play behavior.	Play behavior^1,3^
Parsons (1986b) UK	VI: *N* = 18 Si: *N* = 18 Age:20 m. -4.4 y.	Visually impaired, no additional impairments, sighted	Observational comparative design	Video analysis of play behavior in a free play situation.	Exploration^2,3^ Play behavior^3^ Specific behavior^2^
Perez-Pereira and Conti-Ramsden (2001) Spain	Bl: *N* = 3 (dyads) Si: *N* = 1 (dyads) Age (Time 1: 28-34 m.) (Time 2: 35–40 m.)	Blind, (premature), sighted	Descriptive comparative design	Monthly video analysis of mother child interaction. Focus on maternal directives	Interaction^2^ Joint attention^3^
Preisler (1991) Sweden	Bl: *N* = 7 VI: *N* = 3 Age: 3–12 m.	Blind, visually impaired, no additional impairments (1 premature)	Longitudinal observational descriptive design	Video analysis of mother child interaction in play situation.	Interaction^1,2^ Intersubjectivity^1,2,3^ Joint attention^1,2^ Exploration^1,3^
Preisler (1993) Sweden	*N* = 9 Age: 2–3 y. to 6–7 y.	Blind ( 4 premature)	Longitudinal observational descriptive design	Detailed description of child’s activities and social situation in a group of sighted children	Exploration^1,3^ Specific behavior^4^
Preisler (1995) Sweden	Bl: *N* = 7 Bl (dyads) Deaf: *N* = 7 (dyads) Age: 3 to 9 m. (at start) – 6 y.	Blind, deaf, no additional impairments, 1 mother deaf	Longitudinal observational design	Transcribed video analysis of parent child interaction focus on pre-verbal abilities, exploration of toys, social and symbolic play, communicative intent and sharing of experiences.	Interaction^3^ Intersubjectivity^1,2^ Joint attention^2,3^ Exploration^1,3^ Play behavior^1,3^
Rattray and Zeedyk (2005) UK	*N* = 5 dyads Si M/ Si ch: *N* = 1 VI M/ Si ch: *N* = 1 Si M/ VI ch: *N* = 1 VI M/ VI ch: *N* = 2 Age:6–18 m.	Visually impaired, sighted	Longitudinal observational design	Bimonthly video analysis parent child interactions during free play focus on early dyadic interactions (touch, vocalizations and facial orientation).	Interaction^3^ Joint attention^2^ Exploration^2,3^
Recchia (1998) USA	*N* = 3 (dyads) Age: 20 - 38 m.	Blind, no additional impairments (1 premature)	Quasi experimental observational descriptive design framework	Video analysis in semi structured play sessions with ambiguous stimuli. Focus on communication in response to spontaneous events, both routine and novel.	Interaction^1,3^
Rettig (1994) USA	Age: preschool age	Visually impaired	Review	Characteristics of play of preschool-aged children and suggests areas for interventions to enhance play behaviors	Play behavior^3^
Rogers and Newhart-Larson (1989) USA	*N* = 10 Age: preschool	Blind from Leber’s congenital amaurosis (*N* = 5) or from other causes	Comparative descriptive design	Comparison of two matched control groups ASS and Leber on Reynell Zinkin, CARS, ABC	Interaction^1^ Play behavior^3^ Specific behavior^1,2^
Rogers and Puchalski (1984a) USA	*N* = 16 Age: 18–38 m.	Visually impaired	Descriptive correlational observational design	Reynell Zinkin scales Object permanence tasks Communication of ‘no’ Symbolic measures (paradigm of Bretherton to elicit symbolic action) Focus on: development of symbolic acts, relationship with language and cognitive variables.	Interaction^1^ Play behavior^2,3^
Rogers and Puchalski (1984a) USA	Bl: *N* = 11 VI: *N* = 10 Age: 4–25 m. Si: *N* = 16 Age: 4–25 m.	Blind, visually impaired (6 additional impairments), sighted	Cross sectional observational descriptive design	Video analysis of mother child interactions.	Interaction^2,3^ Intersubjectivity^2,3^
Rogers and Puchalski (1986) USA	Bl: *N* = 5 (dyads) VI: *N* = 5 (dyads) Age: 4–12 m.	Blind, visually impaired, no additional impairments (4 premature)	Cross sectional and longitudinal observational design	Comparing cognitive skills (Reynell Zinkin Scale) with first social smile, focus on characteristics of social smile: reactive of or initiating interaction? Parental behavior to elicit social smile, frequency of social smile during 1e year.	Interaction^1^ Intersubjectivity^1^
Rogers and Puchalski (1988) USA	Bl: *N* = 11 VI: *N* = 9 Age: 4–25 m.	Blind, visually impaired (10 premature)	Longitudinal descriptive design	BSID, Reynell Zinkin Scale, Strange situation and measure for Object Permanence.	Exploration^1^ Play behavior^2^
Rogow (1982) Canada	Bl: *N* = 8 VI: *N* = 2 Age: 15 m. – 7 y.	Blind, visually impaired with additional impairments	Observational descriptive design	Bimonthly video/ audio analysis, weekly checklists. Focus on structured social routine based in nursery rhymes and communicative behavior.	Intersubjectivity^2,3^ Joint attention^3^ Play behavior^3^
Ross and Tobin (1997) UK		Blind	Review	Influence of congenital blindness on motor functions and reaching for sound making stimuli	Exploration^2^
Rowland (1984) USA	*N* = 5 (dyads) Age: 11–32 m.	Blind, 4 with additional impairments	Longitudinal observational descriptive design	Video analysis mother child interactions	Interaction^1,2^ Intersubjectivity^2,3^ Joint attention^2^
Salvo et al. (2001) Italy	*N* = 14 Age = 12–48 m. (calendar age) *N* = 13 Age 12–48 m. (developmental age)	Blind, visually impaired, with additional impairments	Observational comparative descriptive explorative design	Reynell Zinkin, Adapted strange situation and adapted attachment questionnaire for adults. Focus on development of attachment.	Interaction^3^ Intersubjectivity^1^
Schellingerhout et al. (1997) The Netherlands	*N* = 8 Age: 8–24 m.	Blind, no additional impairments	Experimental descriptive design	Exploratory procedures used to explore a gradient surface texture Exploration of gradient texture was examined over 11 weeks’ time.	Exploration^1,3^
Skellenger et al. (1997) USA	*N* = 24 Age: 2.9 to 5.8 y.	Blind, visually impaired, no additional impairments	Observational descriptive design	Play behavior form Play setting assessment form Video analysis, focus on children’s play behavior patterns and interactions	Interaction^1^ Exploration^3^ Play behavior^1^ Specific behavior^1,2^
Smitsman and Schellingerhout (2000) The Netherlands	*N* = 3 Age: 4 y.	Blind	Experimental design	Searching tasks compared using different textures: homogeneous and specially made gradient textures	Exploration^3^
Sousa et al. (2005) Brazil	Bl: *N* = 4 (dyads) Si: *N* = 4 (dyads) Age: 2–6 y.	Blind, no additional impairments, sighted	Observational comparative descriptive design	Observation during free play, focus on occurrence of autistic features in children with congenital blindness	Interaction^2^ Joint attention^2^ Play behavior^3^ Specific behavior^1^
Tadic et al. (2009) UK	Bl: *N* = 16 VI: *N* = 16 Si: *N* = 17 Age: Bl: 15–53 m. VI: 17–36 m. Si: 10–36 m.	Blind, visually impaired, sighted	Observational correlational comparative design	Video analysis of semi structured play situation Reynell Zinkin scale Focus on establishing, maintaining and shifting attention on toys	Joint attention^1^
Tröster et al. A (1991) Germany	*N* = 85 Age: 10 m. – 6 y.	Blind	Descriptive survey design	The Bielefeld parents questionnaire, focus on frequency, duration and typical situations of the occurrence of various types of stereotypic behavior	Specific behavior^1^
Tröster et al. B (1991) Germany	*N* = 85 Age: 5–72 m.	Blind, no additional impairments	Cross sectional comparative design	Bielefeld Parents’ Questionnaire focus on the frequency, duration and typical situations of the occurrence of various stereotypic behaviors in their children	Interaction^1,2,3^ Exploration^1^ Specific behavior^1,2,3,4^
Tröster and Brambring (1992) Germany	Bl: *N* = 22 Si: *N* = 47 Age: Bl: 9–12 m. Si: 12–19 m.	Blind, no additional impairments (15 premature) sighted	Comparative descriptive design	Comparison of scores on Bielefeld test for blind infant and preschoolers 3 subscales; Emotions, social interactions, impulse control.	Interaction^1,3^ Intersubjectivity^2,3^ Joint attention^1^
Tröster and Brambring (1994) Germany	Bl: *N* = 91 Si: *N* = 73 Age: Bl 4 m. - 6 y. Si 4 m. - 4 y.	Blind, (41 premature) sighted	Descriptive survey design	Comparing sighted and blind children Questionnaires parents	Interaction^2^ Exploration^2^ Play behavior^1,3^
Wills (1979) USA	*N* = 2 Age: 0 m. - 3 y.	Blind no additional impairments (2 premature)	Longitudinal observational descriptive design	Observation during weekly (case 1) and fortnightly visits (case 2)	Interaction^1^ Play behavior^3^
Withagen et al. (2010) The Netherlands	*N* = 48 (*N* = 21 Age: < 4 y. )	Blind no additional impairments	Descriptive comparative design	Tactual skills evaluated with tactile profile, divided over several age groups including birth to 2 years and 2–4 years	Exploration^1,3^

Interaction ^1 child’s contribution 2 caregiver’s contribution 3: attunement^

Intersubjectivity ^1 development 2 child’s behavior 3 caregiver’s contribution^

Joint attention ^1 development 2 child’s behavior 3 caregiver’s contribution^

Exploratory behavior ^1 development 2 connection with other developmental domains 3 child’s behavior^

Play behavior ^1 development 2 connection with other developmental domains 3 characteristics^

Specific behavior ^1 connection with VI 2 characteristics 3 connection with other developmental domains 4 circumstances^

*Bl* Blind

*Si* Sighted

*VI* Visually impaired

*According to WHO norms, blind is defined visual acuity <0.05. Different terms, used in the articles: legally blind, with light perception, without light perception, profound visually impaired, no form perception are brought under this term

**Additional impairments. In the articles additional impairment are often not well defined. Mostly cognitive and/or motor impairment, often with neurological background. Auditory impairment is not included under this term but mentioned separately

On the basis of the selected articles in each theme, the first two authors independently distinguished subthemes. Agreement on subthemes was reached afterwards and then discussion in the research group contributed to fine-tuning the definitions of these subthemes. Subthemes, emphasis and conclusions found in the literature and relevant for the adaptation of the video feedback intervention to VIPP-V are described.

## Results

Review of the literature revealed that most studies had small (varying from *N* = 1 to *N* = 91) and heterogeneous samples often including children with severe/profound visual impairments and blindness. The samples usually were not well defined with respect to the visual impairment (e.g., blindness, profound visual impairment). This is due to the variety of definitions in the different countries and regions, now and in the past. Different etiologies and the often unclear nature of additional impairments (e.g. prematurity is usually not mentioned as a risk factor in development) complicate comparison of studies and generalization. Results are therefore presented tentatively and should be interpreted with caution.

Thirty-three studies reported results based on an observational study design compared to only four studies with an (quasi) experimental design. The descriptive studies reported longitudinal data (*k* = 18) or cross-sectional data (*k* = 21). Seven review articles were included. Data was collected most often using video analysis (*k* = 31). Other methods included interviews, questionnaires, scales, and live-observation (Table 1).

### Interaction/Communication

For the theme Interaction/communication 52 articles were included. Three subthemes were distinguished: 1) contribution of the child: nonverbal and verbal signals (*k* = 24); 2) contribution of the parents (*k* = 15); and 3) attunement of parent and child in interaction (*k* = 21).

#### Contribution of the Child

Qualitative and quantitative differences in the nonverbal and verbal signs of children with visual impairments are found compared to sighted children. The visual impairment is hypothesized to influence the nature of signs given by the child, although personal strengths and weaknesses of the child are also deemed important (Recchia 1998).

- Nonverbal signals of the child
  The majority of studies on children with visual impairments describe a limited repertoire of social behavior, limited facial expressions (Baird et al. 1997; Kekelis and Prinz 1996; Parr et al. 2010; Skellenger et al. 1997; Tröster and Brambring 1992; Wills 1979), less or no initiation of affectionate games and less or no reaching out to the mother as an initiative gesture to be picked up (Fraiberg 1975). Only one study mentioned a higher proportion of nonverbal turns in a child with blindness in dialogue (Conti-Ramsden and Perez-Pereira 1999). Infants with profound visual impairments do not smile as frequently as sighted children and it is more difficult to elicit a smile. Smiling is not used to initiate contact; it usually is a response, aimed at maintaining contact (Fraiberg 1968, 1975; Rogers and Puchalski 1986).

Nonverbal signs of children with visual impairments seem to differ not only quantitatively but also qualitatively from the nonverbal signs of sighted children; postural cues may indicate (dis)comfort, the hands can function as an organ for maintaining contact, and ‘quieting’ in babbling can be seen as an indication of stranger anxiety (Fraiberg 1968, 1975). Nonverbal signs are often idiosyncratic, and sometimes of a stereotyped nature (Fazzi et al. 1999).

b. Verbal signals of the child
  There appear to be quantitative and qualitative differences between the verbal signs of sighted infants and infants with a visual impairment. Studies indicate that although there is great variety in patterns of development and in some children, once started, rapid progress in language (McConachie and Moore 1994), children with profound visual impairments are at risk of developmental delay in language (Dale and Sonksen 2002; McConachie and Moore 1994; Wills 1979). In some studies fewer periods of positive vocalizations, fewer responses and fewer initiations were found in the dialogue of children with visual impairments with their caregiver compared to sighted children (Rogers and Puchalski 1984b; Skellenger et al. 1997). Another study however showed that blind children can be equally capable in communication as sighted children, also in initiating and maintaining conversations (Conti-Ramsden and Perez-Pereira 1999).

Some studies reported positive outcomes on specific aspects of language, e.g. the frequency of vocalization within normal limits, although with a lag for a period in the second year for children with blindness (Fraiberg 1968; Rowland 1984). The relation between symbolic play level and language abilities in children with visual impairments is comparable to that of sighted children (Lewis et al. 2000). However, most studies focus on differences in the content of language of children with visual impairments. These qualitative differences are found especially in studies of children with blindness as opposed to studies of children with visual impairments. Infants with blindness vocalize when exploring toys but do not share their experiences (Preisler 1991); sighted peers actively form hypotheses about word meaning while children with blindness are slow to generalize words (Andersen et al. 1984). A delay is seen in the acquisition of the word ‘I’, and is interpreted as a problem of self-representation in play and language (Fraiberg and Adelson 1973). Deviant language (such as echolalia, pronoun reversal, perseverative speech, limited attempts at communication, little use of social speech) is described in children with visual impairments, especially in relation to emotional or behavioral deficits and specific syndromes such as Leber’s congenital amourosis (Hobson et al. 1999; Ophir-Cohen et al. 2005; Rogers and Newhart-Larson 1989; Tröster et al. 1991b).

#### Contribution of Caregivers

Studies report a greater amount of physical and verbal involvement of mothers in the group of children with visual impairments compared to mothers of sighted children (Behl et al. 1996; Conti-Ramsden and Perez-Pereira 1999; Tröster and Brambring 1994). The contributions in interaction in the dyads of mother and child with a visual impairment are not symmetric. This is not due to less input from the child but to more contributions from the mother (Conti-Ramsden and Perez-Pereira 1999; Kekelis and Prinz 1996). Compared to mothers of children with severe visual impairments, mothers of children with blindness are more active in initiating and maintaining interaction (Moore and McConachie 1994; Perez-Pereira and Conti-Ramsden 2001; Rowland 1984).

Parents in interaction with their child with a visual impairment seem to be more directive and to control activities more often than parents of sighted children (Behl et al. 1996; Campbell 2003; Conti-Ramsden and Perez-Pereira 1999; Perez-Pereira and Conti-Ramsden 2001; Preisler 1991; Rowland 1984). Some researchers suggest that this directive interactive style has a negative effect on child development (Kekelis and Andersen 1984; Rogers and Puchalski 1984b; Rowland 1984), other authors suggest that this style can be an appropriate functional response to the special needs that lack of sight imposes (Behl et al. 1996; Conti-Ramsden and Perez-Pereira 1999; Hughes et al. 1999; Perez-Pereira and Conti-Ramsden 2001; Tröster et al. 1991b). Within dyads the directives seem to decrease when the child grows older (Perez-Pereira and Conti-Ramsden 2001) and directive parental involvement is not seen in all dyads (Sousa et al. 2005). Mothers’ responsive behaviors rather than their initiatives have the most impact on the development of children and result in more positive outcomes (Dote-Kwan and Hughes 1994; Dote-Kwan 1995; Dote-Kwan et al. 1997). Mothers of children with visual impairments tend to use a familiar format or repertoire for their interactions (Campbell 2003) and they repeat directives more often (Conti-Ramsden and Perez-Pereira 1999).

#### Attunement

Parents have specific expectations about the development of affect and communication in the interaction with their child (Als et al. 1980). In dyads of parents and sighted children eye contact and voice contact are integral parts in first relationships. It is a challenge for caregivers of children with visual impairments to enable children to understand more about themselves and others in interaction with limited visual input (Campbell 2007). Pre-linguistic communication can be established with non-visual behavior such as touch, vocalizations and facial orientation. Research has shown that dyads in which both parent and child are visually impaired, can engage in sophisticated communicative exchanges prior to infants’ acquisition of language (de Medeiros and Salomão 2012; Rattray and Zeedyk 2005). However, most studies report that caregivers’ interpretation of the child’s behavior is limited (Baird et al. 1997; Mallineni et al. 2006; Preisler 1991; Tröster and Brambring 1992). Subtle signals from children are not always noticed and if behavior is registered by parents (e.g. slight movement of the head or stiff posture), it is not always interpreted as an attention marker (Preisler 1995). Expressive behavior of the child is less frequently reinforced because it is more difficult to recognize differentiated emotions in the child (Tröster and Brambring 1992).

Several studies on children with blindness state that their delay in language acquisition and perspective taking hinders attunement (Andersen et al. 1984; Brambring 2005; Brambring and Asbrock 2010; Farrenkopf and Davidson 1992). Children with blindness have difficulties in just those areas of language acquisition where visual information can provide input about the world and are a stimulus for forming hypotheses about pertinent aspects of the linguistic system (Andersen et al. 1984). In verbal role-play children who are blind often lack the specific ability to understand shifting perspectives (Andersen et al. 1984). The need for caution in explaining autism-like behavior if vision is insufficient to support early social and communicative development in children is emphasized (Parr et al. 2010; Tröster et al. 1991b). Pleasure and positive feedback for each member of the dyad may be reduced because of fewer exchanges of positive vocalizations and responses (Rogers and Puchalski 1984b). However, communication styles and family contexts can influence the interaction positively (Kreutz and Bosa 2009; Recchia 1998).

As in all children the quality of communication is richer with the mother than with a stranger (Tröster and Brambring 1992). However, lack of vision impedes the acquisition of a dialogue concept (Tröster and Brambring 1992) and when another (cognitive, emotional or behavioral) deficit is added to the visual deficiency, the quality of communication diminishes and proves even more compromised (Demingeon-Pessonneaux et al. 2007; Ophir-Cohen et al. 2005). It has been suggested that the absence of vision as an organizer of experience makes it difficult for the child to form a stable mental representation of the attachment figure (Fraiberg 1968). One study found that 60% of children with visual impairments and children with visual and profound intellectual impairments were insecurely attached to their parents (Salvo et al. 2001), but perhaps the parental reaction to the birth of an infant with blindness has a more profound effect on the quality of the attachment relationship than the visual impairment itself (Rattray and Zeedyk 2005).

Thus agreement is found on the nonverbal signs of the child with a visual impairment which appear to differ in quantity and quality; less expressions, a limited repertoire of expressions and different expressions. Less consonance is found in studies on verbal signs. Children with profound visual impairments appear to be at risk of a developmental delay and in children with visual impairments, the content of verbal signs seems to be different from that of sighted children. In interaction with their child, parents tend to take more initiative to initiate and maintain interaction and take a directive controlling role. As the quality of interaction is richer with the parents than with others, interventions to stimulate interaction would be most beneficial if provided to the parents. These interventions should focus on teaching parents how to recognize their child’s nonverbal behavior and reinforce expressive behavior to improve parent-child communication and ultimately improve the quality of parent-child interaction.

### Intersubjectivity

Focusing on the preverbal interaction between infant and caregiver (intersubjectivity) 16 articles were included, the majority of these articles focus on children with blindness only. The following subthemes emerged: 1) development (*k* = 8); 2) child’s behavior (*k* = 11); and 3) caregiver’s contribution (*k* = 9).

#### Development

In the early weeks of life of an infant with blindness there seems no reciprocity yet, but ‘we see a form of tactile seeking in the blind baby’… ‘a brief pursuit of the mother’s hand’… to restore a contact that has momentary lost’ (Fraiberg 1975, p.320). The infant shows awake immobility especially when there is auditory input. This will modify to attentive stillness, reacting with mimic and rhythmical movements to auditory and tactile signals when the infant is three weeks of age. Attending and orienting periods alternate with brief periods of disorganization/time-off (Als et al. 1980).

Mothers respond to the facial expressions and body movements of their infants (Preisler 1991) and from three months of age reciprocity emerges. From that moment on parents and infants get involved in proto conversations: synchronized activities and interaction patterns (Als et al. 1980; Loots et al. 2003; Preisler 1991). The social smile emerges in infants with blindness from the age of four months, but does not occur often and mostly they smile in response (Fraiberg 1968, 1975; Rogers and Puchalski 1986). From the age of six months infants show delight by smiling, cooing, increased motor activity and attempts to ‘talk’ when mothers refer to actions and feelings of the child (Preisler 1991, 1995). Infants also start selective smiling when stranger anxiety emerges (Fraiberg 1968). Although the above-mentioned studies show that intersubjectivity can also develop in children with blindness, one study suggests that visual impairment can impede the development of intersubjectivity and secure attachment (Salvo et al. 2001).

#### Child’s Behavior

In their first weeks of life, infants with blindness are quiet and immobile (Als et al. 1980; Fraiberg 1968). Periods of regression precede new accomplishments. In these periods of regression children exhibit restless, overreacting behavior and are difficult to soothe and unavailable to communication (Als et al. 1980). Between three and six months of age children are more responsive and react with increased motor activity when their mothers approach them (Preisler 1991, 1995).

When mothers play rhythmic body touching games and sing, expectancy awareness is observed (Preisler 1995). Infants demonstrate increased awareness of their role in social interaction. They vocalize and/or gesture in social routines and by positioning their body to indicate the beginning of a social routine (Rogow 1982). Preliminary behaviors for the development of joint attention are described: for instance the child with blindness uses the adult’s body or fingers to find objects, or performs instrumental acts that may be interpreted as gestures concerning the object (Bigelow 2003). Infants with blindness show a limited repertoire of facial expressions, different vocalisations, have no initiative gestures, and a repertoire of nonconventional idiosyncratic gestures. Hand movements are used as a way to communicate (Fraiberg 1975; Rogers and Puchalski 1984b; Rowland 1984).

#### Caregiver Contribution

Eight studies describe contributions of the caregiver in the development of intersubjectivity including five dealing with children with blindness only (Als et al. 1980; Fraiberg 1968; de Medeiros and Salomão 2012; Rowland 1984; Tröster and Brambring 1992). Studies focusing on periods of stillness of the child in the first months and on periods of disorganization, mention the danger of miscommunication and misunderstanding between parents and their children (Als et al. 1980; Fraiberg 1968). Attentive periods in the child alternate with periods of regression; these attentive periods can be easily overlooked and misunderstood (Als et al. 1980).

For parents of a child with a visual impairment the different pattern of responsiveness in vocalizations (Rogers and Puchalski 1984b; Rogow 1982; Tröster and Brambring 1992), and the limited and different repertoire in gestures (Fraiberg 1968; Rowland 1984) compared to sighted children can be difficult to interpret, especially when the child has additional impairments (Baird et al. 1997). It is difficult for parents to perform contingent and consistent reactions to their child’s signals. Their responses to vocalizations are weak and inconsistent (Rogers and Puchalski 1984b; Rowland 1984; Tröster and Brambring 1992). Parents often use too much vocal stimulation, and take too little time to pause or listen (Rowland 1984). They do not always recognize the differentiated emotions of their child, so expressive behavior is less frequently reinforced. Therefore infants with blindness experience less regularly a connection between their own behavior and that of the partner (Tröster and Brambring 1992). In one study it was observed that during the first year of the child’s life, mothers start to use a more directive verbal style and less affect attunement behavior (Preisler 1991). Rearing a child with a visual impairment can contribute to feelings of depression, which can form a danger to intersubjectivity (Fraiberg 1968). Resourceful parents however can learn to understand the distorted signals of their infant (Als et al. 1980) or can use touch and body language to facilitate communication (de Medeiros and Salomão 2012).

In summary, the signals of the child are not always easy for their parents to interpret, especially in very young children with blindness and in children with additional impairments. The child’s periods of stillness and of disorganization together with the different patterns of responsiveness in vocalizations and in gestures can lead to miscommunications. Differentiated emotions in the child are difficult to read and therefore less frequently reinforced by parents. Parents can learn to ‘read’ their child but need help to adequately interpret the signals of their child. This emphasizes the importance of support.

### Joint Attention

In 24 articles joint attention is described, the majority dealing with children with blindness. The following subthemes emerged: 1) development (*k* = 11), 2) child’s behavior (*k* = 7) and 3) caregiver’s contribution (*k* = 9).

#### Development

Where joint attention generally emerges in sighted children between nine and twelve months of age, studies mention a delay in the development of joint attention in children with visual impairments (Bigelow 2003; Preisler 1991). In their first year of life children with visual impairments interact with their caregivers without objects involved. In a longitudinal study of children with severe visual impairments and children with blindness, the two children with moderate/severe visual impairments focused on the outside world and checked back on their mothers’ reactions at nine months of age. The children with blindness interacted with their mothers about toys a few months later, at twelve months of age (Preisler 1991). Pre-joint attention for an activity or object can be initiated by the mother from the age of four and a half month (Als et al. 1980).

Maintaining attention and shifting attention from the mother to the object is more difficult for children with blindness and for children with profound visual impairments compared to sighted children. These attentional components may be the most difficult part to establish in a joint interaction episode (Tadic et al. 2009). Joint attention is also dependent on cognitive development, making the mastery of this skill even more challenging for children with visual-and-intellectual impairments. Understanding the relationship between cause and effect prepares children for understanding intentionality in others. The comprehension of object permanence is necessary for children with blindness to understand that they can obtain and explore objects (Bigelow 2003).

The use of language can facilitate joint attention (Bigelow 2003; Preisler 1991). However, studies that describe the relationship between joint attention and language development mainly refer to the problems of creating and sharing symbolic and linguistic meanings during moments of joint attention (Loots et al. 2003). These studies also refer to the difficulties of children with profound visual impairments to understand language which refers to objects (Dale and Sonksen 2002). Problems of children with visual impairments to comply with simple commands (e.g. ‘show me.’) may be an expression of the difficulty these children experience in including external objects into the interaction with caregivers (Tröster and Brambring 1992).

Joint attention is the first expression of the evolving comprehension that others have intentions (Theory of mind). Perspective taking, the growing ability to understand others perspective, is the first stage (Brambring and Asbrock 2010). Some studies describe a delay in the development of this ability. Children with blindness have limited possibilities to experience that other people are related in a shared world (Farrenkopf and Davidson 1992; Hobson et al. 1999). Other authors who report a delay in the development of perspective taking in children with blindness show that when perspective taking tasks are adjusted for children with blindness, the delay is comparable with delays in other developmental areas (Brambring and Asbrock 2010).

#### Child’s Behavior

Blindness per se may not predispose a child to limited joint attention, but a delay in sharing of experiences about objects with others seems evident (Preisler 1991). The traditional requesting gestures (looking, pointing, reaching), which require visual reference, are not used by children with severe visual impairments and blindness (Preisler 1995; Rowland 1984). In language, problems with perspective taking abilities are reflected (Andersen et al. 1984; Preisler 1991, 1995). Studies mention a different way of expressing a desire to share attention in children with visual impairments: they vocalize more often when exploring toys, which may be an indication of wanting to share experiences (Preisler 1991). From nine months of age children with blindness can occasionally be observed to ‘ask questions’ about sounds: their intonation is question like and they move their head and upper body towards sounds (Preisler 1995). Facial orientation may be less important for communication during periods of shared focus of attention than touch (Rattray and Zeedyk 2005). Although most studies describe a delay and problems in the development of joint attention in children with blindness, one study describes that two out of four dyads of caregiver-child with blindness had a higher frequency of joint attention episodes than the sighted control group. However, the operationalization of the joint attention episode in this study is questionable (Sousa et al. 2005).

#### Caregiver’s Contribution

As children with visual impairments do not use most of the conventional signals, it is difficult for parents to read their preferences (Preisler 1995). Question-like intonation or movements of children with blindness that could occasionally be observed from nine months of age, were not always answered by caregivers because signals were unnoticed or misinterpreted (Preisler 1995). Several studies describe how caregivers not always follow their child’s attention closely (Kekelis and Andersen 1984; Kekelis and Prinz 1996; Moore and McConachie 1994). Mothers tended to introduce a greater proportion of topics that focus on the child rather than on other persons and events in the environment (Kekelis and Andersen 1984), and made fewer references to the objects the child was focused on and more references to potential objects (Moore and McConachie 1994). They asked their children fewer real questions and more test questions while playing (Kekelis and Prinz 1996). On the other hand, several studies describe mothers of children with blindness to be more active in the establishment of joint attention by giving more directives containing descriptions (Campbell 2003; Conti-Ramsden and Perez-Pereira 1999; Perez-Pereira and Conti-Ramsden 2001), or by simultaneously touching the object and maintaining proximity (de Medeiros and Salomão 2012).

To summarize, joint attention not only emerges later in children with visual impairments but these children also show different ways of expressing their desire for joint attention. Traditional requesting gestures are not used. Their vocalizations, their question like intonation and their body language are not always interpreted adequately and answered by their parents. Although studies describe how parents can help their children by giving more directions and descriptions, these parents may need help to closely follow their child’s attention to facilitate joint attention.

### Exploration

The theme exploration included 34 studies, that together describe the following subthemes: 1) development (*k* = 16); 2) connection with other developmental domains (*k* = 13); and 3) child’s behavior (*k* = 16).

#### Development

The majority of studies on the development of exploration include samples of children with severe and profound visual impairments and blindness. Only three studies described meticulously the beginning of the development of exploration in the first months of life (Als et al. 1980; Fraiberg 1968, 1975). Parents play an active role in the development of exploration: they encourage their child to touch objects and teach their child about persons and objects in the outside world (Preisler 1991).

The initial indication of an infant’s awareness of itself in relation to its environment is their reaching for objects on external cues; by reaching they express their understanding of themselves in the physical world with objects which they can explore (Bigelow 2003). Fraiberg (1968) describes in her study the development of reaching in children with visual impairments Reaching begins when exploring the face of intimate persons with fingers and hands. In the first months of life children with blindness do not reach towards objects. However, when a soundless toy is removed from the hands, children with blindness will sometimes produce a fleeting gesture of pursuit. Between six and eight months of age children retrieve an object nearby when it falls from their hand, only immediately after prior tactile experience.

Absence of vision seems a major impediment to localizing sounds. While grasping (maybe awkward and uncertain) follows the developmental timetable, there seems no adaptive substitution for reaching (Fraiberg 1968). Sighted children start reaching to visual clues when they are twelve weeks old. The emergence of reaching on a sound cue in infants with blindness varies between six and eleven months of age (Als et al. 1980), and some children never find an adaptive solution. Fraiberg (1968) speaks of children with ‘blind hands’ (page. 284), who are not able to use their hands for exploration. Reaching on a sound cue requires object permanence skills which children with visual impairments generally master months later than sighted children (Bigelow 1992; Rogers and Puchalski 1988; Withagen et al. 2010). Throwing objects in different directions is a favorite game for children with visual impairments; perhaps this is a strategy to create a concept of space (Preisler 1993).

Exploratory activities change in children with visual impairments when manipulation skills develop: rubbing and fingering emerge from grasping (Smitsman and Schellingerhout 2000). Tactile exploration involves cooperative activity of kinesthesis and cutaneous sense, by which manipulation is adapted to the texture gradient. Rotation movements emerge when shapes change (Landau 1991; Schellingerhout et al. 1997). In comparison with sighted children, children with blindness need more manipulation of objects to explore it’s shape, it takes one and a half times longer to habituate to novel objects and it is more difficult for them to find the movable parts of toys (Landau 1991). Exploration of functional aspects of toys starts at the average age of 15 months (Preisler 1995). An infant with blindness can learn to manipulate and compare objects by devising a strategy involving the simultaneous use of two objects, one in each hand (Gerhardt 1982). In young children with visual impairments exploratory play is their only and dominant play behavior. Introducing other types of play meets with resistance (Celeste 2006; Ferguson and Buultjens 1995; Preisler 1993). The increase in general movement activity in the second year of life seems to be accompanied by an increase of stereotyped behaviors (Tröster et al. 1991b).

#### Connection with Other Developmental Domains

When children grow older, exploration expands due to improving motor skills (Rattray and Zeedyk 2005; Tröster and Brambring 1994): children can hold and explore toys, and more active touch is seen (Parsons 1986b). Manual control is essential for the development of the capacity to classify objects (Gerhardt 1982). Mobility skills seem to be related to explorative behavior; the mastery of crawling seems to advance or occur at the same time as the ability to reach for a sound cue (Bigelow 1992; Fraiberg 1968). It has been suggested that the understanding of space and object is tied to the development of a concept of causality and self-agency (Bigelow 2003). However, recent studies contradict this assumption and have highlighted the importance of exploratory actions for motor, perceptual and cognitive development in infancy. Only through action do children learn about themselves and their environment (De Campos et al. 2012). Exploratory actions are seen as a prerequisite for learning possibilities, which implies that children who experience reduced opportunities to explore may have limited ability to process information and may have less complex exploratory behaviors (De Campos et al. 2012; Fraiberg 1975; Ross and Tobin 1997).

Tactile and movement experiences are well represented in the early words of the child with blindness. Concrete experiences are important for their language development. When children with blindness begin to use multi-word sentences, they can also select an object on the basis of the name (McConachie and Moore 1994). Children with low scores on language and sensory motor understanding and those with emotional and behavioral deficits, exhibit developmental delay in exploratory behavior (Ferguson and Buultjens 1995; Ophir-Cohen et al. 2005).

#### Child’s Behavior

Exploratory behavior of children with visual impairments looks somewhat different from exploratory behavior of sighted children: children with visual impairments play with busy hands (Olson 1983) and a face with little expression (Fraiberg 1968), but vocalize more while exploring (Preisler 1991). Exploring objects often occurs in a repetitive manner, less functional and more stereotypical (Fraiberg 1968; Parsons 1986b; Preisler 1993, 1995; Skellenger et al. 1997), while unfamiliar objects are resisted (Ferguson and Buultjens 1995). The large amount of time children with visual impairments spend in simple manipulative behavior may demonstrate their need for information about (play) materials through channels other than vision (Skellenger et al. 1997). A high proportion of infants and toddlers with blindness show unusual responses to objects. Under- and overreaction to tactile stimuli (Hobson et al. 1999) is mentioned, which inhibits children from exploring objects with their hands (McConachie and Moore 1994). Sometimes children do not bring their hands together in midline (Fraiberg 1968). Many children with visual impairments rather than immediately engaging with the materials, ask questions or make statements about the materials as if they explore by asking questions (Lewis et al. 2000). Language can be used as an exploration tool but as noted above, where visual information can provide input about the world, the use of language referring to visual aspects is difficult (Andersen et al. 1984). In episodes of joint attention with their caregivers children use active touch more often than in solo play (Rattray and Zeedyk 2005). Active touch involves activities of the hands, feet and mouth (Schellingerhout et al. 1997) sometimes clasping the objects against the body (Withagen et al. 2010).

To summarize: exploration of objects is delayed and children with visual impairments need more time to get information on form, structure and function of objects. In exploratory actions children learn about themselves and their environment. Therefore interventions should inform parents on the delayed development of exploration and the unusual repetitive and less functional responses of their child with a visual impairment.

### Play

For the theme play 22 articles were included. The majority of articles focused on play in children with blindness. The following subthemes emerged: 1) development (*k* = 6); 2) connection with other developmental domains (*k* = 6); and 3) characteristics (*k* = 16).

#### Development

Children with visual impairments have a delay in development of play and have quantitative and qualitative differences in their play compared to sighted children on all levels of play. These differences in play widen with age (Parsons 1986a; Tröster and Brambring 1994); where sighted children explore toys from twelve weeks onwards by eye-hand coordination and reaching and grasping, children with blindness start to explore qualities of toys or objects with mouth and hand at around six months of age (Preisler 1995)**.** Games of give and take emerge in children with visual impairments around 13 months of age and exploration of functional aspects of play objects at the average age of 15 months (Preisler 1995). In children with visual impairments, pretend play also develops later: in a longitudinal study only one out of seven children with blindness showed simple pretend play before 18 months of age (Preisler 1995). In younger children exploratory play is the only and dominant play behavior. When children grow older, they show more functional constructive and fantasy play and spend more time with these activities (Ferguson and Buultjens 1995). These findings conflict with the findings of Skellenger et al. (1997) which state that when children grow older they spent less time in interaction with other children and in total play.

Imaginative play with words and sounds starts at around 18 months (Ferguson and Buultjens 1995) and between ages three to four and a half years imaginative play with objects emerges in children with blindness (Fraiberg and Adelson 1973). According to Tröster and Brambring (1994) children with blindness rarely have symbolic play, which in their view does not indicate a delay in representational intelligence but is caused by the unsuitability of traditional toys for symbolic play for these children. Playing with fantasy often takes the form of verbal role-playing (Andersen et al. 1984). Children with blindness are at risk of becoming stuck at lower levels of play and failing to move to more age-appropriate levels. These children may need intervention to facilitate play and enhance their overall development (Skellenger et al. 1997).

#### Connection with Other Developmental Domains

A connection between play, language development (Verbal Comprehension: VC and Expressive language: EL) and Sensory Motor Understanding (SMU; Reynell-Zinkin Scale; Reynell 1979) has been observed (Ferguson and Buultjens 1995). Children with low scores on language and SMU spent more time in exploratory play and resisted the introduction of other types of play. The correlation between frequency and duration of fantasy play and VC and EL was highly significant. (Ferguson and Buultjens 1995). In children with visual impairments imaginative play with dolls emerges between the age of three and four-and-a-half years and corresponds with the use of the self-reference pronouns ‘me’ and ‘I’ (Fraiberg and Adelson 1973). A strong relationship between symbolic acts and the use of the word ‘no’ has also been found (Rogers and Puchalski 1984a). A relationship between object permanence and symbolic play was observed but the onset of symbolic play appears more closely related to other expressions of representational concepts like language development (Rogers and Puchalski 1988). Problems with reversibility are considered to be a manifestation of a general lack in perspective taking (Andersen et al. 1984). The playful context of joint attention behavior is regarded as an indicator of the child’s growing awareness of the adults’ role in play (Bigelow 2003).

#### Characteristics

Higher percentages of exploratory play and functional play than symbolic play have been described in a group of children with blindness compared to a sighted control group. Most children showed some symbolic play but at a lower level than the control group (Sousa et al. 2005). Children with low vision have less functional and more stereotyped play, compared to sighted children (Parsons 1986b).

Several studies focus on the qualitative differences in play between children with blindness and sighted children. Two studies describe the intensity of playful exploration of a toddler with blindness who fingered the objects, rotated them and explored intensively by putting them in her mouth and hand (Gerhardt 1982; Preisler 1995). Children with low vision are less able to engage in sustained play with toys and need more assistance (Kekelis and Prinz 1996). While playing, children ask more questions. Children with low vision find transitions difficult; they resist moving from the known to the unknown (Rettig 1994). An interesting phenomenon in play of children with visual impairments is the ‘play’ with words and sentences. Children with blindness enjoy nonsense words and rhymes; they appear to use speech as an area of play to a far greater extent than sighted children do (Rogow 1982; Wills 1979).

Qualitative differences in fantasy play between sighted children and children with visual impairments have also been found (Parsons 1986a). Andersen and her colleagues (1984) showed that fantasy play of children with blindness is usually verbal role-play in which past conversations between themselves and others are repeated or reconstructed. Tröster and Brambring (1994) however, stated that children with blindness rarely show symbolic play. Perhaps this is because these authors refer to symbolic play with toys. In their imitation of symbolic actions toddlers with visual impairments have fewer schemes, less diversity and fewer sequences than sighted children (Rogers and Puchalski 1984a). Qualitative differences were also observed in social play. The four and a half year old girl mentioned in Celeste’s study (2006) had limited social interactions; she preferred interactions with adults and failed to respond to peers’ advances.

Children with visual impairments can develop the ability to play functionally and symbolically but are limited in demonstrating these skills (Lewis et al. 2000). However, when they have additional impairments their ability to play becomes more limited. Children with blindness and characteristics of autism spectrum disorders (ASD) are closely similar to sighted children with ASD in their diversity of play behavior and play with peers. They diverge however in their development of symbolic play. Seven out of nine children with blindness and ASD had symbolic play compared to two out of nine sighted children with ASD (Hobson et al. 1999). Children with Leber’s Congenital Amaurosis (LCA) compared to a control group of children with congenital blindness, played alone for long periods of time, preferring sameness in handling objects. The repertoire of activities was very limited and they had no symbolic play (Rogers and Newhart-Larson 1989).

These results indicate that children with visual impairments have a delay in development of play and have quantitative and qualitative differences in their play compared to sighted children. Their play is not always recognized as such. Parents need help to adequately observe and facilitate their children’s play behavior.

### Specific Behavior

For this theme 19 articles were included. The following subthemes emerged: 1) connection with visual impairment, syndromes, cognitive impairment (*k* = 10); 2) characteristics (*k* = 9); 3) connections with other developmental domains (*k* = 6); and 4) circumstances and other persons’ reactions (*k* = 8).

#### Connection with Visual Impairment, Syndrome, Cognitive Impairment

Several studies have been conducted regarding the connection between stereotyped specific behavior and visual impairment. Younger children with visual impairments show more stereotyped behavior and have a broader repertoire of this behavior (Brambring and Tröster 1992; Sousa et al. 2005; Tröster et al. 1991a, b). When children grow older, stereotyped behavior diminishes. Only the frequency of eye-poking and body rocking increases from the first to the second year of life and occurs relatively often during the pre-school years. Eye-poking and body rocking are by far the most frequent stereotyped behaviors in children with blindness (Tröster et al. 1991b). Besides age, the degree of the visual loss seems directly related to the frequency of mannerisms: stereotyped behavior diminishes with age but persists longer and is seen more often in children with blindness (tactile learners) than in children with visual impairments (visual learners) (Freeman et al. 1989; Parr et al. 2010; Skellenger et al. 1997).

Visual self-stimulation by eye pressing occurs in children with retinal disorders, but not in children with cerebral visual impairments (Jan et al. 1983). Most vigorous and intense eye pressing is seen in children with retinal disorders such as retinopathy of prematurity and in children with Leber’s congenital amaurosis (LCA). When eyes are totally destroyed or enucleated the eye pressing stops. The occurrence of eye pressing is also dependent on the age of onset of the visual impairment and the degree and quality of the residual vision. A possible explanation for eye pressing is sought in brain functions; the urge to press may be originated in the urge to pass signals from the brain (Jan et al. 1983). An additional explanation of the eye pressing points to the occurrence of autistic like behavior in children with LCA. These children have increasing stereotyped behavior at the end of the second or early third year of life: perseverative and repetitive behavior with little appropriate play (Rogers and Newhart-Larson 1989). Qualitative differences in stereotyped behavior between children with autism and children with visual impairments have been found. The differences seem to lie in intensity, duration and persistence of the stereotyped behaviors (Gense and Gense 1994).

#### Characteristics

The most frequently observed stereotyped behaviors are body rocking (Fazzi et al. 1999; Jan et al. 1983; Tröster and Brambring 1992), and head shaking (Bigelow 2003). Results regarding the frequency of eye pressing and eye poking, repetitive handling of objects, hand and finger movements, lying face down and jumping are less consistent (Fazzi et al. 1999; Jan et al. 1983; Tröster and Brambring 1992). Most children display several stereotyped behaviors (Tröster et al. 1991b). Some stereotyped behaviors occur and quickly disappear; other behaviors are frequent and long lasting. Eye poking and body rocking prove to be relatively stable over time (Brambring and Tröster 1992; Tröster et al. 1991b). Frequency of occurrence seems a predictor for stability of the stereotyped behavior (Brambring and Tröster 1992). The range of stereotyped behaviors decreases from the age of three years up to school enrollment (Tröster et al. 1991b). Repetitive handling of objects is not always described as stereotyped behavior, but repetitive play behavior is common in children with severe visual impairments (Parsons 1986b; Rogers and Newhart-Larson 1989; Skellenger et al. 1997).

#### Connection with Other Developmental Domains

Stereotyped repetitive behavior can be a serious threat for development in children with visual impairments (Fraiberg 1968). The stereotyped behavior often restricts the child’s opportunity for learning and experience, and in some circumstances can even lead to physical injury (Tröster et al. 1991b). Several studies point to the relationship between stereotyped behavior and communicative behavior, but results are inconsistent. Some studies state that stereotyped behavior prevents communication (Fazzi et al. 1999), while others suggest the opposite: that inappropriate communication may lead to handling toys in a stereotyped manner (Kekelis and Andersen 1984) and can be prevented by sensitive interpretation of the child’s signals (Als et al. 1980). This relationship between repetitive stereotyped behavior and communication is supported by several studies which show a significant negative correlation between stereotyped behavior and expressive language; the higher the scores on expressive language the less repetitive behavior is exhibited (Ferguson and Buultjens 1995). In addition to communicative behavior, motor limitations and a reduced capacity for exploration are also related with stereotyped behaviors (Fazzi et al. 1999). However, the question is whether this reduced capacity causes the stereotyped behavior, or vice versa.

#### Circumstances, and Other Person’s Reactions

During critical periods in the development of children with visual impairments, any stimulation seems to produce an overreaction (Als et al. 1980). Stereotyped behavior is usually elicited by excitement, anger or delight. On finding a desired toy for example, the physical activity (flapping of limbs, shaking and pulling behavior) increases. This behavior suggests that infants are modulating the level of stimulation through self-action (comparable to looking away in sighted children when overwhelmed by over-arousal) (Bigelow 2003; Brambring and Tröster 1992; Fazzi et al. 1999). Boredom caused by restricted environmental conditions, reduced sensory stimulation and reduced mobility will also elicit stereotyped behavior (Preisler 1993). Children will for example roll over, lying face down on the floor when objects were taken from them or if the content of the interaction was too abstract. It is assumed that the inability to communicate may lead to this stereotyped behavior (Ferguson and Buultjens 1995).

Situational conditions that can elicit stereotyped behavior seem to change with the child’s age. When the child reaches the primary school age, cognitive and concentrative demands can provoke this behavior. Situations where the child is left and arousal situations can also elicit stereotyped behaviors (Brambring and Tröster 1992; Tröster et al. 1991b). Caregivers often misinterpret this behavior. Headshaking for example is sometimes interpreted as a negative response (Bigelow 2003). Most parents try to stop stereotyped behavior (Campbell 2003) not only because this behavior seems to hinder development, but also because stereotyped behavior is often seen as a sign of mental retardation or psychological disturbance. A display of this behavior may lead to stigmatization (Tröster et al. 1991b).

To summarize: especially in young children with visual impairments a broad pattern of stereotyped behavior is often seen. Some behaviors occur and disappear, but others are frequent and long lasting. Situational and emotional conditions influence the appearance. Expressive language seems to correlate negatively with stereotyped behavior. Intervention should inform parents on stereotyped behavior in children with visual impairments and the most optimal conditions to prevent it. The overall view in the literature strongly supports adjusting interventions such as the VIPP on the six themes. Results as described above on the six themes with their subthemes are summarized in Table 2.

**Table 2:** Themes and subthemes found in the literature on children with visual impairments and the parent-child relationship

Theme	Articles (*k*)	Subthemes (*k*)
Interaction	52	Child’s contribution (24)
		Caregivers’ contribution (15)
		Attunement (21)
Intersubjectivity	16	Development (8)
		Child’s behavior (11)
		Caregivers’ contribution (9)
Joint attention	24	Development (11)
		Child’s behavior (7)
		Caregivers’ contribution (9)
Exploratory behavior	34	Development (16)
		Connection with other developmental domains (13)
		Child’s behavior (16)
Play behavior	22	Development (6)
		Connection with other developmental domains (6)
		Characteristics (16)
Specific behavior	19	Visual impairment, syndrome, cognitive impairment (10)
		Characteristics (9)
		Connections with other developmental domains (6) Circumstances other persons reactions (8)

## Implications for Practice

Strong support is found in the literature for the choice of interventions such as the VIPP that focus on parental sensitivity. The studies that investigate ways to improve interaction between children with visual impairments and their parents also focus on emotional and verbal responsiveness of the parents. Even the amount of time parents spend together with their child in one room is related to the development of expressive pragmatic language and the amount of initiative behavior of the child (Dote-Kwan and Hughes 1994; Dote-Kwan 1995; Rogow 1982; Wills 1979). However, the quality and appropriateness of parental responses, not the quantity, are the most important factors in the stimulation of (verbal) interaction (Hughes et al. 1999). Parental emotional and verbal responsiveness in recognizing and reinforcing nonverbal behavior of the child, their listening skills (Mallineni et al. 2006), their repeating or rephrasing of children’s words, pacing the rate of speech, and length of pauses between turn taking (fine tuning) are all positively related to children’s development of interaction skills (Dote-Kwan and Hughes 1994; Dote-Kwan 1995; Rowland 1984). The results support the importance of the themes mentioned in VIPP: 1) exploration versus attachment behavior, 2) promoting accurate perception of signals of the child 3) explaining the relevance of adequate responding to the signals and 4) sharing emotions. However, some aspects need more attention.

Exploration versus attachment behavior: For exploration additional information is required in intervention on the conditions for play and the different expressions of play in children with visual impairment. To facilitate play in children with visual impairments it is important to limit the number of toys so children can keep track of them (Lewis et al. 2000). The play objects themselves can be more or less stimulating; gradient textures for example are more inviting for children with visual impairments and lead to better results in search tasks during exploration (Smitsman and Schellingerhout 2000). Traditional toys often are not inviting for children with visual impairments (Tröster and Brambring 1994), but common household items with different materials and textures can provide interesting objects to explore, especially when they make a sound (Rettig 1994; Tröster and Brambring 1994). Objects with interesting tactile or auditory effects can stimulate functional play (Parsons 1986a). Most children with visual impairments do not really need objects for symbolic play, as this type of play is usually expressed through sounds or language (Preisler 1993).

To enable parents to notice differences between contact seeking behavior and exploration, descriptions of the necessary conditions for exploration and the specific ways of exploring in children with a visual impairment, need to be provided. The importance of adapting the environment to facilitate exploration and play is mentioned (Rettig 1994). Several studies suggest that structured activities and situations are easier to follow for children with visual impairments and also make transitions smoother when children resist moving from the known to the unknown (Preisler 1993; Rettig 1994). Parents and other caregivers should encourage children to explore objects, facilitate exploration when necessary and teach children about persons and objects in the outside world (Kekelis and Andersen 1984; Kekelis and Prinz 1996; Preisler 1991, 1993). The VIPP should therefore focus on how the parent facilitates exploration.

Accurate perception of and response to the child’s signals: This is a prerequisite to developing intersubjectivity and hence an important focus for interventions with parents. The development of intersubjectivity depends on the provision of a qualitatively rich and varied experience with a parent or caregiver with whom they can experience pleasure and meaning in interaction. Only then will children begin to respond and to elicit a dialogue themselves (Fraiberg 1968). ‘Perfect movement and synchrony’ was seen in children when their mother sang (Preisler 1991, page 76). Expectancy awareness was shown in rhythmic body and touching games (Preisler 1995; Rogow 1982); and while playing these games a turn taking pattern developed (Preisler 1991). Touch, massage and body language are essential to facilitate communication between children with visual impairments and their parents (Lappin and Kretschmer 2005; de Medeiros and Salomão 2012).

To establish joint attention parents need to talk with their children about the object the child is focused on (Bigelow 2003; Preisler 1995). It is important that they refer to the attributes of objects at the child’s current focus of attention and less to potential objects (Moore and McConachie 1994), that they request actions and ask real questions, instead of requests for information (test questions) (Kekelis and Prinz 1996). Social routines based on traditional nursery rhymes are a form of adult-infant interaction through which mutuality and shared attention can be developed (Rogow 1982).

Explaining the relevance of adequate responding to the signals by providing descriptions of specific behavior and advices to diminish these behaviors: This will be crucial in informing and reassuring the parents and will have to be supplemented to VIPP. A stimulating environment can lead to a reduction of stereotyped behavior, and gives children the opportunity to (re)establish contact and communication with the world around them in an appropriate and adaptive way (Fazzi et al. 1999; Tröster et al. 1991a; Tröster et al. 1991b). Situations which induce overstimulation as well as under stimulation should be avoided and children with visual impairments should be offered sufficient time to explore their environment (Fazzi et al. 1999).

It can be difficult to read the emotions of a child with a visual impairment due to limited and different expressions (Baird et al. 1997; Kekelis and Prinz 1996; Parr et al. 2010; Skellenger et al. 1997; Tröster and Brambring 1992; Wills 1979). Attention should be given to reading and differentiating of these emotions. The focus should be on postural cues that may indicate (dis)comfort, anxiety, contact seeking, and joy (Fazzi et al. 1999; Fraiberg 1968; Fraiberg 1975). During the sharing of emotions parents will need to learn to reinforce the difficult to recognize differentiated emotions in the child (Tröster and Brambring 1992).

In general, support of families and reassurance about parental skills are the foundations for healthy development in children. If parents are in grief and worry about the diagnosis of visual impairment in their child, the interactions with their child are more constrained (Kekelis and Andersen 1984). Should there be a setback in the development of the child or in interaction patterns, parents need to be reminded and supported to keep perspective on the process of development and if necessary go back to previous successful ways of interaction (Als et al. 1980).

Early intervention is a practical low-threshold way to support parents and children with visual impairments. Parent-child interaction is an appropriate context for intervention, because of the potential to influence both affective and structural qualities of interaction (Campbell 2007). In intervention parents can learn to pay more attention to movement, facial orientation and vocalizations of their child, to actively touch their child and to listen and be patient in interaction (Loots et al. 2003; Rattray and Zeedyk 2005; Rowland 1984; Tröster and Brambring 1992). Early intervention can be essential to prevent cycles of misinterpretation between parents and their infants with visual impairments (Baird et al. 1997).

In stimulating parent-child interaction the focus should not be on quantitative aspects, but on qualitatively rich and varied experiences (Fraiberg 1968). Synchronized and reciprocal interaction routines help infants to predict their own and others’ behaviors (Kekelis and Andersen 1984; Loots et al. 2003). Infant massage training can stimulate reciprocity in the parent-child interaction and was found to increase more reciprocity in the infant’s reactions (Lappin and Kretschmer 2005). Social routines based on traditional nursery rhymes and familiar story telling can also help children with visual impairments. Moreover, these routines help parents to recognise weak signals of the child as social signals and reinforce these signals so the child will begin to experience interaction (Rogow 1982).

## Discussion

The purpose of this study was to find themes that are important in adapting interventions, in particular the VIPP, that focus on the quality of the relationship between parents and children with visual impairments. The clinical child psychologists and early intervention specialists of Royal Dutch Visio and Bartiméus who participated in a Delphi-study mentioned six themes. Practice-based knowledge was used to determine the themes and provide a focus for the literature search. By doing so, the expertise of the professionals in the field could be combined with a scientific systematic review of the literature. The six themes covered important issues in early parent child relationships: interaction, intersubjectivity, joint attention, exploration, play, and specific behavior. The literature search based on these themes resulted in a large number of articles with a solid representation of all themes mentioned in the Delphi-study. Although the themes ‘exploration’, ‘play’ and ‘specific behavior’ were covered well, most research was focused on the themes ‘interaction’, ‘intersubjectivity’ and ‘joint attention’. The focus on these themes indicates the importance of evolving communication between children with visual impairments and their parents and the evolving interaction between these children and their surrounding world. Not only are these themes important to incorporate in a version of VIPP for children with visual impairments, they can also be applied more widely to inform effective interventions in parent-child relationships.

A caveat for the recommendations and implications of the reviewed work regards the small size and heterogeneity of the study samples. The term ‘visually impaired’ encompasses both legal blindness and partial sight. Studies have often failed to distinguish between children with blindness and those who have some residual vision, thereby masking potential differences. Moreover, there is sometimes a lack of clarity about the presence of additional (especially neurological) impairments. The course of development in children with visual disabilities may be influenced by etiology of the visual impairment, visual function and the presence of co-occurring disabilities (Hatton et al. 1997). This should be taken into account before generalizing the research findings on all six themes.

A visual impairment in itself influences how caregivers and children interact, although functionally, parents and children may overcome the limitations posed by the visual impairment by compensating with the other senses. Compensation not only depends on the abilities of the child but also on the abilities and skills of the parents (Dote-Kwan 1995; Dote-Kwan et al. 1997; Hughes et al. 1999; Mallineni et al. 2006; Rowland 1984). Parents of a child with a visual impairment need to be extra sensitive, that is heightening the alertness in all their senses, to notice their child’s signals, to interpret these signals and to closely follow the child in their responses. Some parents need help to respond in a sensitive way to their children. For these parents, an intervention like VIPP-V may be developed. This applies particularly to parents of children with visual impairments who, in the early stages of the development of their child, have to cope with and accept the impairment of their child. This stress influences their confidence as a parent and in turn has a negative impact on the quality of the parent-child relationship (Tröster 2001).

Besides support for early caregiver-child interaction, parents also may require support concerning the functional aspects of the development of children with visual impairments. For example, they need to know how to stimulate exploration, as exploring the world seems less attractive for these children. Lack of exploration is often influenced by a delayed motor development. Furthermore, qualitative differences in exploration are reported between children with visual impairments and sighted children. Children with visual impairments show unusual reactions to (play) objects compared to children without an impairment: they have more repetitive and less functional behavior and more stereotyped behavior (Fraiberg 1968; Hobson et al. 1999; Parsons 1986b; Skellenger et al. 1997). With regard to play, a delay in play activities in children with visual impairments as well as quantitative and qualitative differences compared to sighted children have been reported (Moleman et al. 2011). Stereotyped behavior is not unusual in children with visual impairments and even common in children with blindness (Brambring and Tröster 1992; Tröster et al. 1991a, b). In general, children with visual impairments are less active (Ferguson and Buultjens 1995; McConachie and Moore 1994). Thus, the already existing Video Intervention to promote Positive Parenting (VIPP) needs to be adapted to meet the needs of children with visual impairments, who follow a different developmental path than sighted children.

A review of the literature indicated that much can be gained for children with visual impairments when the focus in intervention is on a sensitive parent-child interaction, as it may be done VIPP-V. To optimize the development of adaptive functioning, parents have to be able to notice and interpret the sometimes distorted signals of their child. Preisler’s observations of early communication patterns in infants with blindness compared with children with deafness show that absence of visual information, even more than absence of auditory information, reduces opportunities to learn and understand interpersonal rules in communication, relations between objects and symbols as well as knowledge about the environment (Preisler 1995). Therefore, adequate and appropriate parent-child interaction will help the child with a visual impairment to develop additional ways of communicating.

Early intervention may be highly useful for children with visual impairments in order to avoid maladaptive development from very early stages. The predominant developmental issue of early infancy is the establishment of social relationships that children may learn to trust and rely on, and the development of these and new relationships continue to be predominant developmental themes. Secure parent- child relationships may be at least as important for the quality of life of children with visual impairments as for other children, and may be an important resilience factor for the risks facing (psychosocial) development in children with visual impairments. The VIPP program, with its focus on sensitivity and attachment, has proven to be effective in many studies, yielding effect sizes that average well above those found across intervention programs in general (*d* = .47 for k = 12 studies for VIPP, compared to *d* = .34 overall; Juffer et al. 2016). By leveraging this solid basis for intervention and incorporating the themes identified in this review, parents and children might be offered a new intervention that will support them through the challenges of early development.
